# Classification of mental workload using brain connectivity and machine learning on electroencephalogram data

**DOI:** 10.1038/s41598-024-59652-w

**Published:** 2024-04-21

**Authors:** MohammadReza Safari, Reza Shalbaf, Sara Bagherzadeh, Ahmad Shalbaf

**Affiliations:** 1 Institute for Cognitive Science Studies, Tehran, Iran; 2 Department of Biomedical Engineering, Science and Research Branch, Islamic Azad University, Tehran, Iran; 3 Department of Biomedical Engineering and Medical Physics, School of Medicine, Shahid Beheshti University of Medical Sciences, Tehran, Iran

**Keywords:** Mental workload, EEG, Brain connectivity, Feature selection, Computational neuroscience, Biomedical engineering

## Abstract

Mental workload refers to the cognitive effort required to perform tasks, and it is an important factor in various fields, including system design, clinical medicine, and industrial applications. In this paper, we propose innovative methods to assess mental workload from EEG data that use effective brain connectivity for the purpose of extracting features, a hierarchical feature selection algorithm to select the most significant features, and finally machine learning models. We have used the Simultaneous Task EEG Workload (STEW) dataset, an open-access collection of raw EEG data from 48 subjects. We extracted brain-effective connectivities by the direct directed transfer function and then selected the top 30 connectivities for each standard frequency band. Then we applied three feature selection algorithms (forward feature selection, Relief-F, and minimum-redundancy-maximum-relevance) on the top 150 features from all frequencies. Finally, we applied sevenfold cross-validation on four machine learning models (support vector machine (SVM), linear discriminant analysis, random forest, and decision tree). The results revealed that SVM as the machine learning model and forward feature selection as the feature selection method work better than others and could classify the mental workload levels with accuracy equal to 89.53% (± 1.36).

## Introduction

Mental workload (MWL) is a concept that refers to how hard the brain is working to meet task demands^1^. It refers to the number of cognitive resources required to perform a task and can be influenced by various factors such as task complexity, time pressure, and environmental conditions. It is a complicated, person-specific, dynamic, and non-linear construct that is multidimensional^2^. Some theories have been suggested to define, explain, and measure MWL, but a single reliable and valid framework to measure it has not been established yet. The MWL can have adverse effects on workability, and identifying and optimizing the factors affecting MWL and workability is crucial^3–5^. The measurement of MWL is important for both science and human factors. From a scientific perspective, quantifying MWL allows researchers to predict operator and system responses, optimize human–machine interactions, and determine the sources of error to enhance work performance in various industries, including medicine^6^. This is crucial for the development of effective strategies to manage MWL and improve task performance. From a human perspective, understanding and managing MWL is essential for maintaining well-being and preventing the negative effects of excessive mental demands, such as stress, fatigue, and performance decrements. Therefore, the measurement of MWL plays a vital role in both scientific research and the improvement of human work conditions and performance^2^.

There are many methods for measuring MWL, including information processing studies, time-line analysis, operator activation level studies, subjective questionnaires, physiological measures, and modeling^7^. The abundance of measurement methods for MWL can result in inconsistent results, and there is currently no consensus on a specific method suitable for all applications. Despite these challenges, the measurement of MWL remains a crucial aspect of scientific research and human factors as it allows researchers to predict operator and system responses, optimize human–machine interactions, and determine the sources of error to enhance work performance. The subjective measures are the most commonly used method for measuring MWL, as they are low-cost easy to administer, and have a high degree of face validity. A common method is to use a questionnaire asking subjects to rate the difficulty of the task^6^. There are some well-known indices like the National Aeronautics and Space Administration (NASA) Task Load Index (NASA-TLX)^8^ and Subjective Workload Assessment Technique (SWAT)^9^. However, they are subject to bias and may not accurately reflect the actual MWL^10^. Measuring MWL through performance measures involves evaluating individuals' task performance as an indirect indicator of their MWL. This approach assesses how well individuals perform a task to infer their cognitive workload^11^.

Finally, the psychophysiological measures method assesses MWL by analyzing physiological signals like heart rate variability, EEG, and fNIRS^12^. Among these, EEG is extensively used due to its quick data acquisition, convenient usage, real-time assessment, lack of subject bias, portability, high temporal resolution, and non-invasiveness^13,14^. Traditionally, machine learning methods based on EEG have been used to classify MWL classes using various algorithms, like support vector machine (SVM)^15–23^, and linear discriminative analysis (LDA)^24–27^. These methods involve feature extraction, feature selection, and classification of EEG signals to measure MWL. Recently, in^28^ a framework for assessing MWL is proposed. This framework uses discrete wavelet transform (DWT) to decompose the EEG signal for extracting the non-stationary features of task-wise EEG signals. Additionally^29^, aimed to investigate the cognitive workload of fighter pilots during different flight phases using physiological signals such as ECG and EEG. The researchers employed classification algorithms, including LDA, SVM, and k-nearest Neighbor (KNN), to classify the pilots' cognitive workload. The results demonstrated that LDA and SVM, with an accuracy of 75%, were more consistent classifiers compared to the k-NN classifier, which achieved an accuracy of 60%. Another study aimed to investigate the impact of theta-to-alpha and alpha-to-theta band ratios on creating models capable of discriminating self-reported perceptions of MWL. The study utilized the STEW dataset and found that models trained with high-level features extracted from the alpha-to-theta ratios and theta-to-alpha ratios achieved high classification accuracy. This indicates the richness of information in the temporal, spectral, and statistical domains extracted from these EEG band ratios for the distinction of self-reported perceptions of MWL^30^.

Many studies have used various methods to assess MWL. However, there is still no single robust method that can accurately assess the MWL. To tackle this issue, we are exploring a brain connectivity-based approach that has shown promising results in various domains^31–33^. So, we will use neural activity flow based on the direct directed transfer function (dDTF) to explore different regions and networks that distinguish between different levels of MWL. Additionally, we will utilize neural activity flow as a feature in machine learning (ML) models such as SVM, LDA, Decision Tree (DT), and Random Forest (RF) to classify MWL levels. Finally, we will use the feature selection method to select features from all frequency bands and filter out irrelevant or redundant variables to improve the accuracy of the model. This approach aims to enhance the understanding of neuronal mechanisms underlying MWL. The main novelties and contributions of our study are the use of dDTF as a measure of effective neural connectivity for the purpose of extracting features from EEG data and proposing a hierarchical feature selection method to select the most significant features, and finally investigating some ML models and compare their results.

## Material and methods

### Participants and EEG recording

We utilized the Simultaneous Task EEG Workload (STEW) dataset, an open-access collection of raw EEG data from 48 male subjects who participated in a multitasking workload experiment utilizing the SIMKAP multitasking test. The SIMKAP multitasking assessment involves participants in identifying and marking identical items across two panels, all while answering auditory questions that vary in type, such as arithmetic, comparison, or data retrieval. Certain auditory tasks may necessitate delayed responses, prompting individuals to monitor a clock positioned in the upper right corner. This multitasking segment follows a predetermined sequence of questions^34^. By focusing solely on male participants, the dataset minimizes variability arising from gender-related physiological differences that could impact EEG data collection and analysis. This approach allows for a more controlled examination of mental workload patterns and EEG responses, particularly in multitasking scenarios like those assessed in the SIMKAP experiment. The experiment consisted of two stages:

1. Information was gathered for 2.5 min when the participants were not engaged in any activity, referred to as ‘low’ MWL.
2. The participants took the SIMKAP test while their brain activity was monitored, and the last 2.5 min were considered the high MWL condition.

The EEG signals were obtained using the Emotiv EPOC EEG headset, featuring a 16-bit A/D resolution, and 128 Hz sampling frequency. Also, 14 channels including AF3, F7, F3, FC5, T7, P7, O1, O2, P8, T8, FC6, F4, F8, and AF4 based on the 10–20 international system, in addition, CMS and DRL were as reference channels. The STEW dataset is valuable for studying multitasking workload and analyzing brain activity during different cognitive tasks. Researchers can use this dataset to develop and evaluate algorithms and models for MWL classification and prediction.

### Preprocessing

We implemented the preprocessing pipeline recommended in the database-providing paper^34^. The pipeline involved:

1. High-pass filtering of the raw data at 1 Hz to filter out low frequency noise that can come from sources such as movement of the head and electrode wires, perspiration on the scalp, or slow drifts in the EEG signal over many seconds
2. Removing line noise which is caused by electrical equipment, such as power lines, that emit electromagnetic fields that interfere with the EEG signal
3. Performing Artifact Subspace Reconstruction (ASR) to automatically detect and remove unusual noise or artifacts from EEG signals
4. Re-reference the data to average to transform the data from a fixed or common reference to an 'average reference,' which is advocated by some researchers, especially when the electrode montage covers nearly the whole head

The application of ASR was emphasized due to the presence of large amplitude artifacts in the data. ASR is a non-stationary method to remove large-amplitude artifacts^35^. The preprocessing was conducted using the EEGLAB toolbox in MATLAB software (version 2019a).

### Effective connectivity

Effective connectivity refers to the directional or unequal dependencies between distinct brain regions^36^. The primary technique for assessing effective connectivity is Granger causality (GC), which can be calculated within the frequency domain. To accomplish this, it is necessary to estimate the parameters of a Multi-Variable Auto-Regressive (MVAR) model for each individual signal dataset. Two crucial parameters for estimating the MVAR model from EEG signals are the window length and the model order. The window length is determined using the Variance-Ratio Test to maintain the stationarity of EEG signals. Subsequently, the estimated model is validated based on the whiteness of residuals, consistency percentage, and stability, and is chosen based on the Akaike Information Criterion (AIC). For a set of M channels of EEG data with lengths of T, denoted as X = {x_1; x_2;…;x_T}, the MVAR process of order p is represented as follows^37^:

1 $${x}_{t}=v+\sum_{k=1}^{p}{A}_{k}{x}_{t-k}+{u}_{t}$$

where $v$ represents an (M × 1) vector comprising intercept terms, denoted as $v={\left[{v}_{1}\dots {v}_{M}\right]}{\prime}$, ${A}_{k}$ are (M × M) matrices of model coefficients, and ${u}_{t}$ signifies a white noise process characterized by a zero mean and a non-singular covariance matrix Σ.

Rearranging terms results in:

2 $${u}_{t}=\sum_{k=0}^{p}{\widehat{A}}_{k}{x}_{t-k}$$

where $${\widehat{A}}_{k}=-{A}_{k}$$ and $${\widehat{A}}_{0}=-I$$.

After applying the Fourier transform to both sides:

3 $$U\left(f\right)=A\left(f\right)X\left(f\right)$$

where

4 $$A\left(f\right)=\sum_{k=0}^{p}{\widehat{A}}_{k}{e}^{-i2\pi fk}$$

By multiply Eq. (4) at $A(f{)}^{-1}$ and rearrange terms we have:

5 $$X\left(f\right)=A(f{)}^{-1}U\left(f\right)=H\left(f\right)U(f)$$

where $X\left(f\right)$ is the (M × M) spectral matrix of the multivariate process, $U(f)$ is a random sinusoidal shocks matrix and $H\left(f\right)$ is the transfer matrix of the system. The spectral density matrix of the process is determined as follows:

6 $$S\left(f\right)=X\left(f\right)X(f{)}^{*}=H(f)\Sigma H(f{)}^{*}$$

The matrices of $S\left(f\right), A(F)$ and $H(f)$ are utilized to establish various metrics of effective connectivity. dDTF explicitly captures directional interactions between brain regions, distinguishing between driving and response regions. This directional information is valuable for understanding the flow of information within neural networks. Also, dDTF allows for frequency-specific analysis of directed connectivity, providing insights into how different frequency bands contribute to information processing within the brain. This can be particularly useful in studying cognitive processes that are associated with specific frequency ranges. In addition, dDTF has proven efficacy in neuroscience investigations^31,32^^37,38^.

The calculation of dDTF from channel j to channel i at frequency f is determined by the following equation:

7 $${dDTF}_{ij}=\frac{|{H}_{ij}(f){|}^{2}}{\sum_{f}{\sum }_{k=1}^{M}|{H}_{ik}(f){|}^{2}}\times \frac{{\widehat{S}}_{ij}\left(f\right)}{\sqrt{{\widehat{S}}_{ii}\left(f\right){\widehat{S}}_{jj}\left(f\right)}}$$

where H(f) is the transfer matrix of the system at a specific frequency and S(f) is the spectral density matrix. We extract five frequency bands for the dDTF measure by averaging the frequency spectrum as follows: delta (2–4), theta (4–8), alpha (8–13), beta (13–32), and gamma (32–50). All steps for dDTF measurement are done in MATLAB software by the Source Information Flow Toolbox (SIFT) version 0.1a^36^.

### Feature Selection

Feature selection plays a crucial role in the interpretability of machine learning models. By carefully choosing which features to include in the model, researchers and practitioners can enhance the understanding of how the model makes predictions. In the context of MWL assessment from EEG data, feature selection is essential for understanding the relationship between brain regions and MWL assessment. By selecting the most relevant EEG features, researchers can better understand the underlying mechanisms of mental workload and provide more accurate and interpretable models. The removal of less important features can help enhance the performance of classification tasks. In order to distinguish between high-MWL and lo-MWL groups, a series of steps were followed. Initially, one-seventh of the data was set aside for testing. Subsequently, the area under the curve (AUC) for each neural activity flow in every band was calculated using LDA. The AUC-ROC value was derived from the mean values of all cross-validation sets, serving as a valid measure of the model's performance in a generalized setting. Following this, the top 30 connections from each frequency band with the highest AUC were selected which made the top 150 features among five frequency bands based on the AUC of each feature, and feature selection algorithms were subsequently applied. This process was designed to leverage neural activity flow features in each band to differentiate between high-MWL and lo-MWL, with a specific focus on the connections exhibiting the highest AUC values. Some popular feature selection algorithms include forward feature selection, minimum-redundancy-maximum-relevance (mRMR), and Relief-F which we have used in this paper. Forward Feature Selection is a stepwise feature selection method that starts with an empty feature set and iteratively adds one feature at a time based on the classifier performance. The process begins by evaluating the individual predictive power of each feature and selecting the best feature. Subsequently, additional features are sequentially added, with each subsequent feature chosen to maximize the improvement in model performance^39^. mRMR algorithm selects features based on their individual and combined predictive power, aiming to build models that capture the most important aspects of the data. It focuses on reducing redundancy and increasing relevance, ensuring that the selected features are both relevant to the problem and non-redundant to each other^40,41^. Relief-F is an unsupervised feature selection method that evaluates the importance of features based on their ability to distinguish between different classes. It measures the decrease in class separation (distance) between the closest neighbors of different classes when a feature is removed. The features with the highest decrease in class separation are selected as the most relevant features^42,43^.

### Classification

In this paper, we’ve used four classifiers for data classifications which are SVM, LDA, Decision tree (DT), and Random forest (RF). SVM is a supervised machine learning model used for classification and regression tasks. It works by finding a hyperplane that separates the data points with the largest margin. SVM is particularly useful for handling both linear and nonlinear input spaces and can be more accurate than other algorithms in certain cases^44^. LDA is an algorithm used for dimensionality reduction and data visualization. It is a probabilistic model that aims to find a linear combination of input features that can maximize the separation between different classes. LDA is commonly used in various applications, such as sentiment analysis and spam detection^45^. A decision tree in machine learning is a supervised learning algorithm that creates models for classification and regression tasks. It uses a tree-like structure where each internal node represents a decision based on an attribute, leading to leaf nodes that represent outcomes. Decision trees are interpretable and widely used due to their simplicity and effectiveness in predicting values based on input features^46^. RF is an ensemble learning method used for both classification and regression tasks. It works by constructing multiple decision trees and combining their predictions to improve overall accuracy. RF is known for its simplicity, scalability, and performance in various applications^47^.

### Statistical analysis

**Figure 1:**
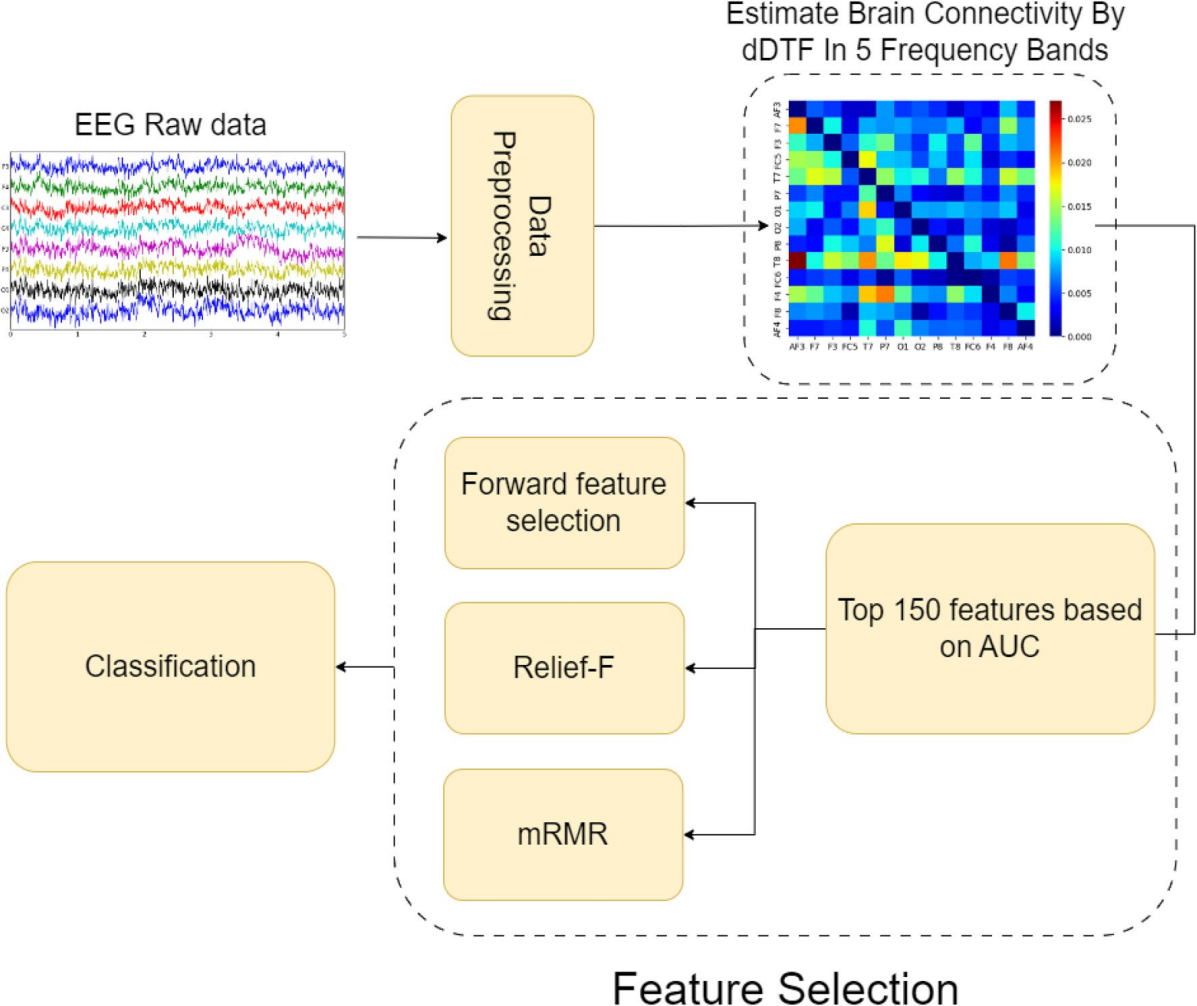
The flowchart of the proposed method. In this method firstly we preprocessed the raw EEG data, then calculated the effective connectivity with the dDTF index in 5 frequency bands. In the next step, the top 30 features based on AUC in each frequency band were calculated and then we applied three feature selection algorithms on them. Finally, we classified the final selected features from each feature selection algorithm.

In our research, we have utilized k-fold cross-validation, a statistical method commonly used in machine learning to estimate the skill of a model. A cross-validation procedure is used to assess the effectiveness of machine learning models, and it can also be used to evaluate a model if there is insufficient data. For cross-validation to be performed, a portion of the training data must be set aside for evaluation later. We partitioned the data into k equally sized segments and then performed k iterations of training and validation. During each iteration, one of the k segments was held out as the test set, while the model was trained on the remaining data. This process was repeated for each segment, and the performance of the model was evaluated and averaged over the k iterations. After conducting a trial-and-error analysis, it was determined that 7 is the optimal value for k. Further analysis was based on the results of the sevenfold cross-validation. The flowchart of the proposed method is provided in Fig. 1. In addition, we applied AUC to selecting the most important connections. The AUC measure, commonly used in evaluating the performance of binary classification models, does not rely on specific assumptions about the underlying data distribution. Instead, it assesses the ability of a classifier to distinguish between positive and negative instances across all possible decision thresholds. However, while AUC itself does not make assumptions about the data, its interpretation can be influenced by certain factors related to the classification problem and the data being analyzed. AUC assumes that the observations used to evaluate the classifier are independent of each other. Violations of this assumption, such as autocorrelation or clustering of observations, can potentially bias the AUC estimate. In addition, AUC is designed for binary classification tasks where there are two distinct classes (which in our case is low-MWL and high-MWL). It may not be directly applicable to multi-class classification problems without appropriate modifications.

## Results

**Figure 2:**
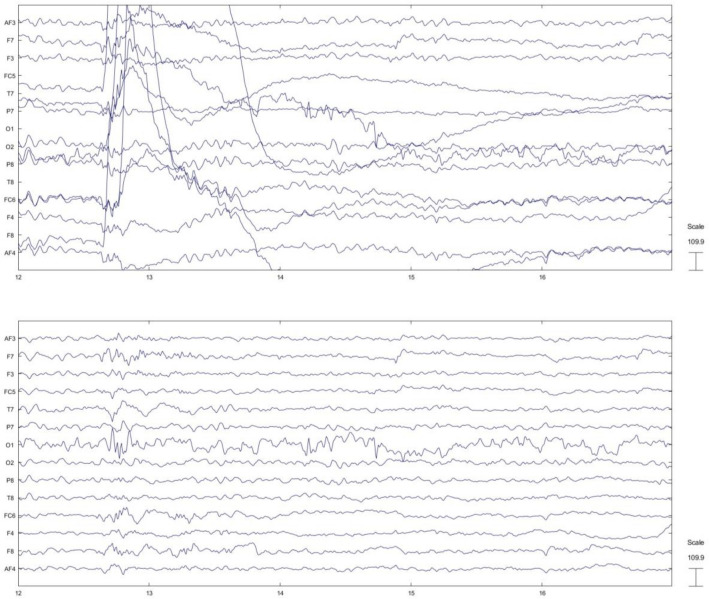
EEG signal before and after preprocessing. The top panel shows the raw EEG signal, while the bottom panel shows the same signal after preprocessing. The preprocessing steps applied to the signal are described in the Methods section.

**Figure 3:**
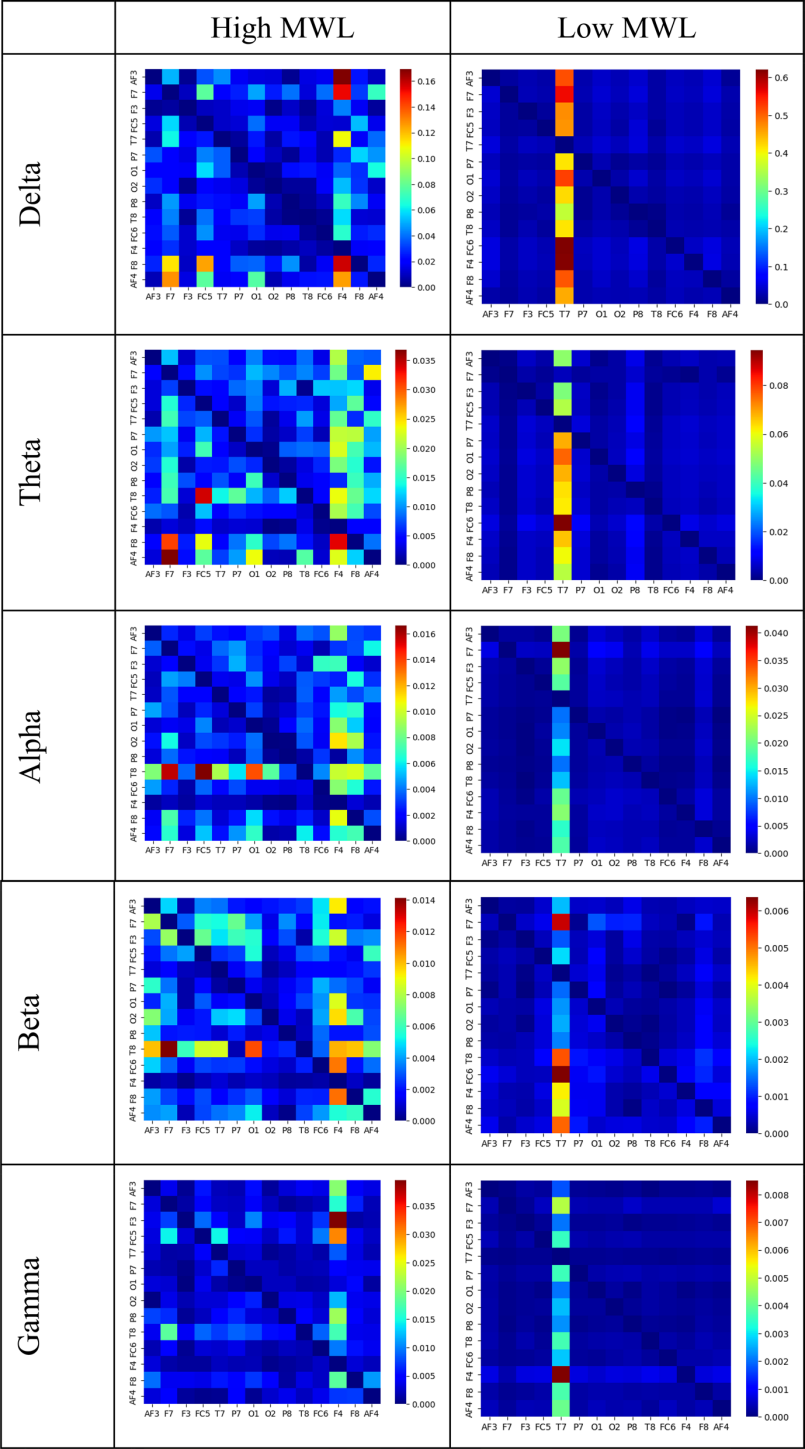
The effective brain connectivity matrices calculated by dDTF for subject-16 in delta, theta, alpha, beta, and gamma bands.

**Figure 4:**
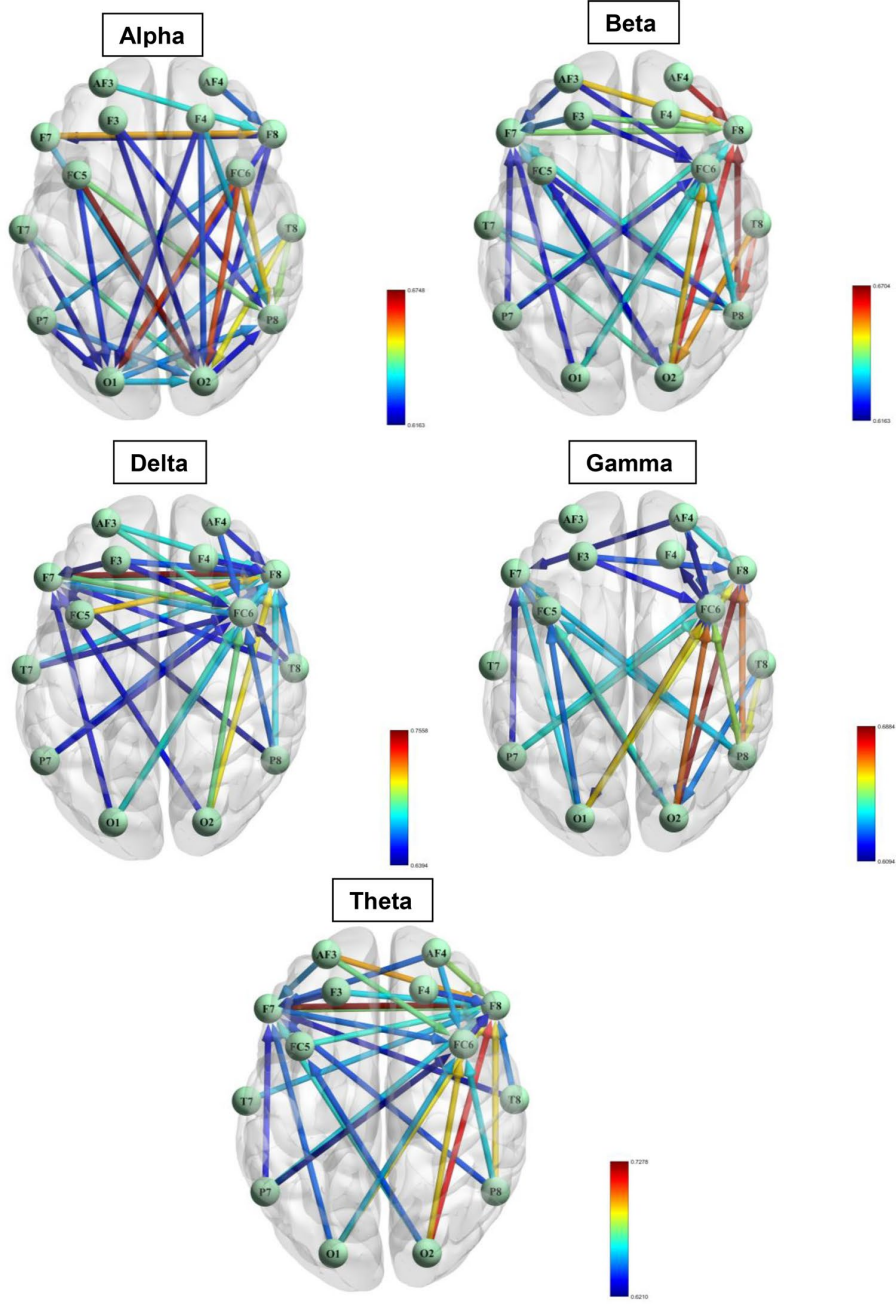
Based on the AUC values, the top 30 neural activity patterns that demonstrate differences in propagation between the high MWL and low MWL groups are depicted. In this illustration, nodes represent electrodes in the 10–20 system, the edges indicate connection between channels, and the edges’ color represent the AUC values.

The EEG data from each subject’s 14 channels were pre-processed using the EEGLAB toolbox in MATLAB software (version 2019a). A sample of data before and after the preprocessing pipeline is provided in Fig. 2. Subsequently, we computed the effective connectivity across all EEG data utilizing dDTF. The dDTF connectivities were derived from sequential 6-s segments of data from 14 channels for each subject across 5 frequency bands. Specifically, a 6-s window was slid along the EEG signals with a step size of 4 s. Figure 3 shows some samples of the dDTF image extracted from ‘high’ and ‘low’ MWL related to subject 16 for each frequency band. Horizontal axes and vertical axes represent channels. Considering 150 s of EEG signals, 6-s as window size, and 4-s as window step, we achieved 37 dDTF matrices per EEG data. Subsequently, the AUC values for each directed connection were computed based on their respective dDTF values. These AUC values are then independently ranked, and the top 30 connections are identified according to their AUC values (Table 1, Fig. 4). In addition, Table 3 provides the number of connections within each region. Based on this table, the frontal lobe has the highest number of neural connections. The classification results and computational efficiency of four machine learning models (SVM, LDA, RF, and DT) for each frequency band and a combination of the top 30 AUC-based features from all bands (top 150) are shown in Table 4. The SVM and DT models demonstrated the best and weakest performance respectively, as indicated in Table 4. In addition, the top 150 features have the highest accuracy, specificity, sensitivity, and F1-measure in all models. Based on the information provided in Table 4, the RF model was the most time-consuming of the four investigated models, while the LDA model was the least time-consuming. We applied a hierarchical feature selection in this paper. So, after selecting 150 top features based on AUC, we used three different feature selection algorithms in parallel including Relief-F, forward feature selection, and mRMR. According to Table 5, the forward feature selection’s results were better than others and the SVM model could achieve 89.53% accuracy on 41 features that were selected based on the forward feature selection algorithm. The 41 selected features based on the forward feature selection are provided in Table 6.

**Table 1:** Based on the AUC values of high-MWL and low-MWL groups, the 30 most significant neural activity flows in the delta, theta, alpha, beta, and gamma bands are as follows.

Delta		Theta		Alpha		Beta		Gamma	
Connection	AUC	Connection	AUC	Connection	AUC	Connection	AUC	Connection	AUC
F7⇒F8	0.756	F7⇒F8	0.728	FC5⇒O2	0.675	P8⇒F8	0.670	O2⇒F8	0.688
FC5⇒F8	0.717	O2⇒F8	0.716	FC6⇒O1	0.663	AF4⇒F8	0.666	P8⇒F8	0.670
O2⇒F8	0.713	AF3⇒F8	0.697	FC6⇒O2	0.663	O2⇒F8	0.666	O2⇒FC6	0.669
O2⇒FC6	0.695	P8⇒F8	0.691	F7⇒F8	0.658	T8⇒P8	0.662	O1⇒FC6	0.661
F7⇒FC6	0.695	O2⇒FC6	0.690	FC6⇒P8	0.654	T8⇒O2	0.655	O2⇒F7	0.660
F8⇒F7	0.692	O1⇒F8	0.688	T8⇒O2	0.652	O2⇒FC6	0.654	O1⇒F8	0.658
AF3⇒FC6	0.687	AF4⇒F8	0.677	T8⇒P8	0.647	AF3⇒F8	0.652	T8⇒P8	0.657
O1⇒FC6	0.686	F8⇒F7	0.675	FC5⇒P8	0.645	F7⇒F8	0.643	P8⇒FC6	0.652
AF3⇒F8	0.684	AF3⇒FC6	0.672	T7⇒O2	0.642	F3⇒F8	0.643	FC6⇒P8	0.648
P8⇒F8	0.679	FC5⇒F8	0.664	AF3⇒F8	0.639	FC6⇒O2	0.642	O2⇒FC5	0.642
T7⇒F8	0.679	O2⇒F7	0.662	O1⇒O2	0.635	T7⇒O2	0.640	P8⇒F7	0.642
FC6⇒F8	0.677	P8⇒FC6	0.659	F4⇒P8	0.635	P7⇒F8	0.638	P7⇒FC6	0.641
O1⇒F8	0.674	F3⇒F8	0.657	FC6⇒P7	0.633	O1⇒FC6	0.638	AF4⇒F8	0.638
FC6⇒F7	0.673	P7⇒F8	0.656	F7⇒O2	0.633	O1⇒F8	0.636	P7⇒F8	0.637
T8⇒F8	0.669	T7⇒F8	0.654	T8⇒O1	0.633	P8⇒F7	0.636	O1⇒F7	0.636
P8⇒FC6	0.663	O1⇒FC6	0.653	O1⇒P8	0.631	FC6⇒P8	0.636	P8⇒FC5	0.634
P7⇒F8	0.662	AF4⇒FC6	0.651	P7⇒O2	0.630	P8⇒FC6	0.636	F7⇒P8	0.633
AF4⇒FC6	0.661	AF3⇒F7	0.649	AF4⇒F8	0.628	T7⇒P8	0.632	T8⇒O2	0.628
F3⇒F8	0.660	T8⇒F8	0.646	F4⇒O2	0.626	O2⇒F7	0.632	O1⇒FC5	0.627
AF4⇒F8	0.655	F7⇒FC6	0.644	FC5⇒O1	0.624	F3⇒F7	0.630	F7⇒O2	0.626
T8⇒F7	0.653	AF4⇒F7	0.643	F3⇒P8	0.623	AF3⇒F7	0.626	AF4⇒FC6	0.624
P7⇒FC6	0.653	O1⇒F7	0.643	F8⇒O1	0.623	FC6⇒O1	0.626	FC6⇒O2	0.624
O2⇒F7	0.652	O2⇒FC5	0.642	O2⇒P8	0.622	P7⇒FC6	0.624	F3⇒F8	0.623
F4⇒F8	0.651	P8⇒F7	0.640	F4⇒O1	0.621	O1⇒F7	0.622	F3⇒FC6	0.618
O1⇒F7	0.650	F4⇒F8	0.640	P7⇒O1	0.620	FC5⇒O2	0.621	F8⇒P8	0.618
F3⇒FC6	0.647	F3⇒F7	0.637	F3⇒O2	0.620	FC5⇒P8	0.621	P7⇒F7	0.614
T7⇒FC6	0.646	P7⇒F7	0.633	F8⇒O2	0.619	F3⇒FC6	0.620	AF4⇒F7	0.611
F3⇒F7	0.644	FC6⇒F8	0.626	O1⇒O1	0.619	P7⇒F7	0.620	FC6⇒O1	0.609
T8⇒FC6	0.642	T8⇒F7	0.625	T7⇒O1	0.617	AF3⇒FC6	0.619	FC6⇒F4	0.609
P8⇒F7	0.639	P7⇒FC6	0.621	F8⇒F7	0.616	O2⇒FC5	0.616	FC6⇒AF4	0.609

## Discussion

In this study, we investigated a new method for the classification of low MWL and high MWL from 14-channel EEG data in 48 participants. In the proposed model we extracted features from EEG signals by the brain’s effective connectivity. In this state, we had 37 matrices with dimensions of 14*14 for each EEG data. For the purpose of extracting the most significant connectivities at the first step, we calculated AUC for all connectivities and selected the top 30 connectivities with high AUC scores which provided 150 features in 5 frequency bands. According to Tables 2 and 3, the most significant connectivities in order to differ between high MWL and low MWL classes are from the frontal lobe. This result is in line with the findings of previous studies^48–50^, who also reported similar outcomes. Table 4 provides a comparison between all features in each frequency band and the top 150 selected features. It revealed that the best accuracy achieved from the top 150 selected features on SVM is equal to 88.96%. At the next step of the feature selection, we applied three feature selection methods on the top 150 features which were forward feature selection, Relief-F, and mRMR. Finally, we used four machine learning algorithms, SVM, LDA, DT, and RF in sevenfold cross-validation to classify the data. Using cross-validation in our research provided a more accurate estimate of out-of-sample accuracy, prevented overfitting, and allowed for more efficient use of data. After the next layer of feature selection, the number of selected features decreased and Table 5 provides a comparison between the accuracy of each algorithm’s results which indicates that the forward feature selection algorithm was most successful among all three feature selection algorithms. Forward feature selection selected the 41 most significant features that are provided in Table 6 and these selected features could achieve an accuracy of 89.53% in SVM which was even better than the accuracy of the top 150 selected features based on AUC.

**Table 2:** An overview of electrodes across all frequency bands is presented.

Channels name	Number of connections from	Channels name	Number of connections to
O1	16	F8	43
O2	16	FC6	29
FC6	15	F7	28
P7	13	O2	17
P8	13	P8	15
F3	12	O1	10
T8	12	FC5	5
AF4	10	AF4	1
AF3	9	F4	1
F7	9	P7	1
FC5	7		
T7	7		
F8	6		
F4	5		

On the left, the electrode names and the number of originating connections are listed, while on the right, the electrode names and the number of connections to which the connections end are provided.

**Table 3:** A comprehensive overview of the connections across different frequency bands in the different regions of the brain.

Region	From						To					
Frontal (AF3, F7, F3, FC5, FC6, F4, F8, AF4)	Delta	Theta	Alpha	Beta	Gamma	Sum	Delta	Theta	Alpha	Beta	Gamma	Sum
	14	14	**19**	13	13	*73*	**30**	**30**	4	21	22	*107*
Occipital (O1, O2)	Delta	Theta	Alpha	Beta	Gamma	Sum	Delta	Theta	Alpha	Beta	Gamma	Sum
	6	7	4	7	8	32	0	0	18	5	4	27
Parietal (P7, P8)	Delta	Theta	Alpha	Beta	Gamma	Sum	Delta	Theta	Alpha	Beta	Gamma	Sum
	5	6	2	6	7	26	0	0	8	4	4	16
Temporal (T7, T8)	Delta	Theta	Alpha	Beta	Gamma	Sum	Delta	Theta	Alpha	Beta	Gamma	Sum
	5	3	5	4	2	19	0	0	0	0	0	0

The table presents the number of connections within each region, including the central, frontal, occipital, parietal, and temporal lobes among five brain frequency bands (Delta, Theta, Alpha, Beta, and Gamma). The 'From' columns indicate the number of connections originating from a specific region, while the 'To' column shows the number of connections ending in the specified region. For instance, there are 19 connections from the frontal lobe in the alpha frequency band, and in total from all five frequency bands there are 73 connections that originated from the frontal lobe. The highest number of connections among all lobes and frequency bands in each group (“From” and “To”) are bold.

Values of significant regions are in [italic].

**Table 4:** A comparison of SVM, LDA, RF, and DT classification results in the delta, theta, alpha, beta, gamma, top150, and top30 bands.

Band	Model	Accuracy	Precision	Recall	F1-Measure	Time consumed (second)
delta	SVM	75.51% (± 2.95)	74.19% (± 3.04)	78.26% (± 5.26)	76.09% (± 3.37)	4.43
	LDA	72.64% (± 3.59)	74.29% (± 3.73)	69.12% (± 5.45)	71.56% (± 4.38)	0.01
	RF	75.00% (± 2.02)	74.09% (± 1.50)	76.75% (± 4.35)	75.36% (± 2.76)	5.11
	DT	67.45% (± 2.09)	68.64% (± 3.14)	64.61% (± 2.99)	66.48% (± 2.03)	0.05
theta	SVM	76.18% (± 2.76)	77.21% (± 3.19)	74.28% (± 3.96)	75.68% (± 3.17)	4.26
	LDA	71.29% (± 2.95)	74.04% (± 3.43)	65.37% (± 5.06)	69.38% (± 4.08)	0.03
	RF	73.76% (± 1.47)	73.07% (± 1.92)	75.33% (± 2.51)	74.15% (± 1.58)	6.38
	DT	65.43% (± 3.15)	66.64% (± 3.13)	62.10% (± 2.73)	64.26% (± 2.67)	0.06
alpha	SVM	80.80% (± 1.46)	81.95% (± 2.21)	79.04% (± 2.74)	80.43% (± 1.74)	4.28
	LDA	76.35% (± 2.27)	74.76% (± 2.55)	79.64% (± 2.92)	77.09% (± 2.25)	0.01
	RF	81.36% (± 2.03)	82.91% (± 2.96)	79.01% (± 2.37)	80.89% (± 2.26)	5.03
	DT	72.86% (± 2.12)	73.04% (± 2.60)	72.64% (± 2.81)	72.79% (± 1.93)	0.06
beta	SVM	82.49% (± 2.21)	82.97% (± 3.64)	81.88% (± 3.85)	82.33% (± 2.54)	4.35
	LDA	78.04% (± 1.02)	78.28% (± 1.53)	77.73% (± 3.47)	77.94% (± 1.36)	0.02
	RF	81.65% (± 1.66)	82.04% (± 2.45)	81.07% (± 1.57)	81.54% (± 1.67)	5.61
	DT	70.38% (± 2.44)	71.34% (± 3.77)	68.37% (± 4.13)	69.72% (± 2.91)	0.06
gamma	SVM	79.56% (± 1.95)	79.90% (± 3.31)	79.29% (± 1.05)	79.54% (± 1.34)	4.23
	LDA	76.58% (± 2.09)	77.78% (± 3.07)	74.68% (± 2.34)	76.14% (± 1.66)	0.01
	RF	79.73% (± 1.71)	79.59% (± 1.81)	80.11% (± 3.26)	79.80% (± 1.61)	5.32
	DT	68.63% (± 2.01)	69.14% (± 2.76)	67.44% (± 1.80)	68.25% (± 1.92)	0.06
top150	SVM	**88.96% (± 1.03)**	**89.10% (± 2.42)**	**88.85% (± 1.64)**	**88.94% (± 1.11)**	**3.91**
	LDA	81.87% (± 1.75)	82.12% (± 1.79)	81.55% (± 3.32)	81.79% (± 1.88)	0.01
	RF	85.81% (± 1.20)	85.84% (± 1.65)	85.81% (± 2.68)	85.79% (± 1.34)	4.85
	DT	75.51% (± 1.48)	76.44% (± 2.55)	74.04% (± 3.42)	75.12% (± 1.40)	0.06
top30	SVM	79.34% (± 1.98)	78.02% (± 2.08)	81.81% (± 4.22)	79.79% (± 2.22)	3.71
	LDA	77.14% (± 2.90)	77.66% (± 2.95)	74.18% (± 4.36)	76.87% (± 3.29)	0.01
	RF	80.24% (± 2.61)	79.18% (± 2.30)	82.08% (± 3.28)	80.58% (± 2.57)	4.65
	DT	72.46% (± 3.10)	73.17% (± 3.27)	71.09% (± 5.69)	71.99% (± 3.60)	0.06

Significant values are in bold.

**Table 5:** A comparison of SVM, LDA, RF, and DT classification results in the delta, theta, alpha, beta, and gamma bands using mRMR, Relief-F and forward feature selection using features from all frequency bands.

Feature Selection Method	Model	Accuracy	Precision	Recall	F1-Measure
Forward Feature Selection	SVM	**89.53% (± 1.36)**	**89.49% (± 2.13)**	**89.65% (± 2.42)**	**89.54% (± 1.36)**
	LDA	83.22% (± 1.40)	83.37% (± 2.05)	83.09% (± 4.26)	83.14% (± 1.84)
	RF	87.50% (± 0.94)	87.93% (± 1.52)	86.93% (± 2.05)	87.41% (± 1.15)
	DT	79.84% (± 2.01)	80.08% (± 2.66)	79.51% (± 2.16)	79.77% (± 1.97)
mRMR	SVM	88.96% (± 0.96)	89.09% (± 2.26)	88.85% (± 1.82)	88.94% (± 1.04)
	LDA	82.09% (± 1.87)	82.53% (± 1.63)	81.42% (± 3.32)	81.94% (± 2.05)
	RF	86.59% (± 1.53)	87.07% (± 2.25)	86.03% (± 3.29)	86.49% (± 1.73)
	DT	75.84% (± 1.37)	76.93% (± 1.75)	73.99% (± 2.24)	75.39% (± 0.97)
Relief-F	SVM	88.96% (± 1.03)	89.10% (± 2.42)	88.85% (± 1.63)	88.94% (± 1.10)
	LDA	82.03% (± 1.64)	82.71% (± 1.68)	81.11% (± 3.61)	81.84% (± 1.77)
	RF	86.37% (± 1.67)	86.24% (± 2.48)	86.59% (± 2.57)	86.38% (± 1.81)
	DT	76.18% (± 0.92)	77.25% (± 1.98)	74.33% (± 1.72)	75.72% (± 0.77)

Significant values are in [bold].

**Table 6:** Top 41 features selected among top-150 features based on forward feature selection and SVM model.

Feature Number	Feature (from⇒to)	Frequency Band	ACC updated by adding this feature to previous	AUC
1	F7⇒F8	delta	0.686	0.756
2	FC6⇒P8	alpha	0.751	0.654
3	F8⇒F7	theta	0.773	0.675
4	FC6⇒P8	gamma	0.791	0.648
5	FC5⇒O2	alpha	0.801	0.675
6	AF3⇒FC6	theta	0.815	0.672
7	T7⇒O2	beta	0.827	0.640
8	O2⇒FC5	theta	0.837	0.642
9	O1⇒F7	gamma	0.845	0.636
10	FC5⇒P8	beta	0.850	0.621
11	O1⇒P8	alpha	0.855	0.631
12	F3⇒F8	beta	0.862	0.643
13	F7⇒F8	beta	0.865	0.643
14	P7⇒F8	gamma	0.868	0.637
15	P7⇒F7	gamma	0.869	0.614
16	FC6⇒O2	gamma	0.874	0.624
17	F3⇒FC6	delta	0.877	0.647
18	O2⇒F7	beta	0.880	0.632
19	FC6⇒O2	beta	0.881	0.642
20	AF3⇒FC6	delta	0.882	0.687
21	F3⇒F8	gamma	0.883	0.623
22	F8⇒O2	alpha	0.883	0.619
23	AF4⇒FC6	theta	0.884	0.651
24	FC6⇒O1	gamma	0.885	0.609
25	FC6⇒F7	delta	0.886	0.673
26	AF3⇒FC6	beta	0.887	0.619
27	O1⇒F7	delta	0.887	0.650
28	T7⇒O1	alpha	0.888	0.617
29	P7⇒F8	theta	0.888	0.656
30	O2⇒F8	gamma	0.888	0.688
31	P8⇒FC6	delta	0.889	0.653
32	T7⇒O2	alpha	0.889	0.642
33	F7⇒FC6	theta	0.890	0.644
34	F4⇒F8	theta	0.891	0.640
35	T8⇒F8	delta	0.891	0.669
36	F3⇒F8	theta	0.892	0.657
37	AF3⇒F7	theta	0.893	0.649
38	O1⇒F8	beta	0.894	0.636
39	F3⇒F8	delta	0.894	0.660
40	P8⇒F7	delta	0.894	0.692
41	O2⇒FC6	delta	0.895	0.695

The proposed framework to classify MWL from EEG data could achieve high accuracy and be in the top range of accuracy between other studies that used machine learning methods to classify MWL into two classes (Table 7). By leveraging brain effective connectivity analysis through dDTF and employing hierarchical feature selection alongside various machine learning models, also with finding and visualizing most important regions in brain for MWL assessment by calculating the brain connectivities, the research significantly has advanced the field of MWL assessment. This approach not only refines the precision of MWL assessment but also contributes to the development of more robust and interpretable models for MWL assessment. In this research, we had some limitations, especially in the dataset. We used the STEW dataset which is a well-known dataset in this field, but this dataset has some constraints such as the low number of participants and gender limitations because the dataset has only male participants and these may affect the generalizability of the findings to the broader population. In addition, the current study used just four machine learning algorithms, but future researchers in this field can try to apply more machine learning algorithms or even deep learning models. Future studies could benefit from expanding the dataset to include a more diverse and representative sample, encompassing participants of different genders and demographics. This would improve the robustness and applicability of the research findings. In addition, researchers can explore a wider range of machine learning algorithms beyond the four used in the current study. Incorporating more algorithms, including advanced deep learning models, can provide a more comprehensive analysis and potentially uncover additional insights from the data.

**Table 7:** Comparison between previous studies which proposed a framework to assess MWL from STEW dataset with two class by machine learning methods.

References	Year	Feature Extraction	Feature Selection	Classifier	Accuracy (%)
^34^	2018	Frequency (Power Spectral Density)	Neighborhood Component analysis (NCA)	support vector regression (SVR)	79.5
^51^	2020	Not specified	Not specified	RF	58.52
^51^	2020	Not specified	Not specified	KNN	61.08
^52^	2020	Frequency features Linear features Non-Linear features	Grey Wolf Optimizer	RF	83
^52^	2020	Frequency features Linear features Non-Linear features	Grey Wolf Optimizer	SVM	83.33
^13^	2021	Graph features	Non-parametric Wilcoxon test	SVM	89.6
Our study	2023	Effective connectivity (dDTF)	Top-AUC + Forward feature selection	SVM	89.53

## Conclusion

In this paper, we proposed a framework for classifying MWL into two classes. To reach this purpose, we used effective brain connectivity as the feature extraction technique and applied four machine learning algorithms including SVM, LDA, RF, and DT. In addition, we investigated a hierarchical feature selection method. In the first step, we extracted the top 30 features based on AUC in each frequency band and then applied three feature selection algorithms which were forward feature selection, Relief-F, and mRMR. The results of this study suggest that machine learning algorithms especially SVM and the proposed framework for feature extraction and hierarchical feature selection can classify MWL levels from EEG data with high accuracy (89.53%). In future investigations, deep learning models can be utilized to construct a robust framework for evaluating MWL through the use of effective brain connectivity images.

## Data Availability

The data used in this study is the Simultaneous Task EEG Workload (STEW) dataset, an open-access collection of raw EEG data from 48 male subjects who participated in a multitasking workload experiment utilizing the SIMKAP multitasking test^34^. The raw dataset is available for download via: https://ieee-dataport.org/open-access/stew-simultaneous-task-eeg-workload-dataset. The data are available to qualified investigators for purposes of scientific research.
